# Stereocomplexation, Thermal and Mechanical Properties of Conetworks Composed of Star-Shaped l-Lactide, d-Lactide and ε-Caprolactone Oligomers Utilizing Sugar Alcohols as Core Molecules

**DOI:** 10.3390/polym9110582

**Published:** 2017-11-06

**Authors:** Kaito Sugane, Hayato Takahashi, Toshiaki Shimasaki, Naozumi Teramoto, Mitsuhiro Shibata

**Affiliations:** Department of Life and Environmental Sciences, Faculty of Engineering, Chiba Institute of Technology, 2-17-1, Tsudanuma, Narashino, Chiba 275-0016, Japan; umintyu.sk@gmail.com (K.S.); rrsgw280@yahoo.co.jp (H.T.); shimasaki.toshiaki@it-chiba.ac.jp (T.S.); teramoto.naozumi@it-chiba.ac.jp (N.T.)

**Keywords:** star-shaped polymer, polymer network, sugar-alcohol, glycerol, erythritol, xylitol, sorbitol, polylactide, poly(ε-caprolactone), stereocomplex

## Abstract

It is important to develop tailor-made biodegradable/biocompatible polymer networks usable for biomaterials whose thermal and mechanical properties are easily controlled by changing the composition. We synthesized sugar-alcohol-based polymer networks (SPN-*m*scLAO/3CLO, *m* = 4, 5 or 6) by the crosslinking reactions of erythritol, xylitol or sorbitol-based *m*-armed star-shaped l-lactide and d-lactide oligomers (H*m*SLLAO and H*m*SDLAO), a glycerol-based 3-armed star-shaped ε-caprolactone oligomer (H3SCLO) and hexamethylene diisocyanate (HDI) at the weight ratios of H*m*SLLAO/H*m*SDLAO = 1/1 and (H*m*SLLAO + H*m*SDLAO)/H3CLO = 100/0, 75/25, 50/50, 25/75 or 0/100). The influence of the arm number on the crystallization behavior, thermal and mechanical properties of SPN-*m*scLAO/3CLOs were systematically investigated by comparing with those of sugar-alcohol-based homochiral polymer network (SPN-*m*LLAO, *m* = 4, 5 or 6) prepared by the reaction of H*m*SLLAO and HDI. Stereocomplex (sc) crystallites are dominantly formed for SPN-*m*scLAO/3CLOs 100/0–25/75, whereas SPN-*m*LLAOs were amorphous. The higher order of melting temperature of sc-crystals for SPN-*m*scLAO/3CLOs 100/0–25/75 was *m* = 5 > *m* = 6 > *m* = 4. The sc-crystallinities of SPN-4scLAO/3CLOs 100/0–50/50 were significantly lower than those of SPN-*m*scLAO/3CLOs 100/0–50/50 (*m* = 5 and 6). The larger order of the sc-spherulite size at crystallization temperature of 110 °C was *m* = 5 > *m* = 6 > *m* = 4 for SPN-*m*scLAO/3CLO 100/0. The size and number of sc-spherulites decreased with increasing crystallization temperature over the range of 110–140 °C and with increasing CLO fraction. Among all the networks, SPN-5scLAO/3CLOs 75/25 and 50/50 exhibited the highest and second highest tensile toughnesses (21.4 and 20.3 MJ·m^−3^), respectively.

## 1. Introduction

**Scheme 1:**
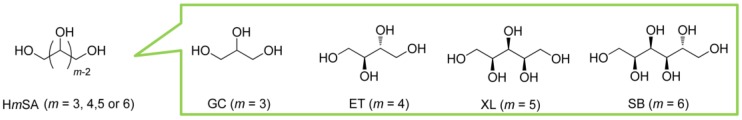
Sugar alcohols (H*m*SAs, *m* = 3, 4, 5 and 6) used in this study.

Star-shaped polymers are branched polymers with at least three macromolecular chains (arms) connected to a central core. Compared with linear analogs of equal molecular weight, they have a higher concentration of functional end groups, and often exhibit lower viscosities, different thermal and mechanical properties and improved physical processability, as their properties are more influenced by arm length than the total molecular weight. Recently, a number of researchers have focused on star-shaped biodegradable/biocompatible polyesters for biomedical applications, including drug delivery, gene delivery, tissue engineering, diagnosis and medical devices [1,2,3]. Until now, a variety of star-shaped biodegradable polyesters have been synthesized by the ring-opening polymerization of cyclic esters such as lactide (LA) [4,5,6,7], ε-caprolactone (CL) [8,9,10,11,12,13], β-butyrolactone [14,15] and trimethylene carbonate [16,17]. Especially, polyesters derived from LA are gathering attention as renewable resources-derived polymers (i.e., bio-based polymers). The ring-opening polymerizations of these monomers have been initiated by various polyhydroxy initiators, such as pentaerythritol, trimethylol propane, dipentaerythritol and tripentaerythritol. However, since these initiators used as cores of the star-shaped biodegradable polyesters are not favorable natural products, there is a possibility that these residual core compounds are not bioresorbed after the complete biodegradation of the polyester arms [18]. Thus, active research on star-shaped biodegradable polyesters possessing sugar alcohols (H*m*SAs) such as glycerol (GC), erythritol (ET), xylitol (XL) and sorbitol (SB) as the core molecules has been carried out in recent years (Scheme 1) [18,19,20,21,22,23]. GC is mainly produced as a by-product in the conversion of naturally occurring fats and oils to fatty acids or fatty acid methyl esters for biodiesel [24,25]. ET, XL and SB occur naturally in some fruits, and are mainly produced by the following processes: ET is produced from glucose by fermentation [26]; XL is produced through chemical reduction of xylose derived from birchwood chips and sugarcane baggase hemicellulose hydrolysate [27]; SB is produced by the hydrogenation of glucose [24,28]. Hao et al. reported the preparation and crystallization kinetics of star-shaped biodegradable poly(l-lactide)s (PLLAs) initiated with GC, ET, XL and SB, and it was found that the more arms of a star-shaped PLLA finally resulted in a lower spherulite growth rate [18]. Xie et al. reported the crystallization and biodegradation of star-shaped poly(ε-caprolactone)s (PCLs) with GC, ET and XL cores, and found that the melting temperature and crystallization rate decreased with increasing arm number [19]. Teng and Xu et al. reported that the crystallinity of star-shaped PLLAs with a XL core decreased with increasing XL fraction, and the biodegradability conversely increased [21]. Teng and Nie et al. reported the controlled drug-release behavior of star-shaped PLLAs with a SB core [22]. 

Recently, network polymers derived from terminal functionalized star-shaped biodegradable polyesters have gathered considerable attention since the curing reaction of their at least three functional groups easily generates crosslinked structures and the crosslinking density can be controlled by changing the arm length [3,13,29,30,31,32,33,34,35,36,37,38]. For example, Storey et al. reported the networks prepared by the crosslinking reaction of hydroxy-terminated 3-armed random copolymers of d,l-LA and CL with l-lysine diisocyanate [30]. Amsden et al. reported biodegradable elastomeric networks prepared by the photo-crosslinking reaction of acrylate-terminated GC-based 3-armed random copolymers of LA and CL [31]. Chang et al. reported the networks prepared by the crosslinking reaction of methacrylate-terminated pentaerythritol-based 4-armed star-shaped l-lactide oligomers [34]. Jahandideh et al. reported the thermomechanical properties of cured products of methacrylate-functionalized star-shaped lactide oligomers with a XL core and their biocomposites with jute fibers [36]. Also, we reported pentaerythritol-based polymer networks (PEPN-4scLAO/2CLOs) prepared by reactions of methylenediphenyl 4,4′-diisocyanate (MDI), hydroxy-terminated two enantiomeric 4-armed star-shaped lactide oligomers bearing a pentaerythritol core (PE4LLAO and PE4DLAO) and a diethylene glycol-based hydroxy-terminated 2-armed ε-caprolactone oligomer (H2CLO) [37], and glycerol (GC)-based polymer networks (GCPN-3scLAO/2CLOs) by reactions of hexamethylene diisocyanate (HDI), hydroxy-terminated two enantiomeric 3-armed star-shaped lactide oligomers (GC3LLAO and GC3DLAO) bearing a GC core and H2CLO [38]. The PEPN-4scLAO/2CLOs and GCPN-3scLAO/2CLOs exhibited relatively high stereocomplex (sc) crystallinity and superior tensile toughnesses due to the incorporation of ductile H2CLO segments. This result is marked contrast to the fact that the corresponding homochiral networks are amorphous. In the past studies, no systematic study on the influence of the arm number of star-shaped polymer networks on the sc-crystallization behavior and physical properties. 

**Scheme 2:**
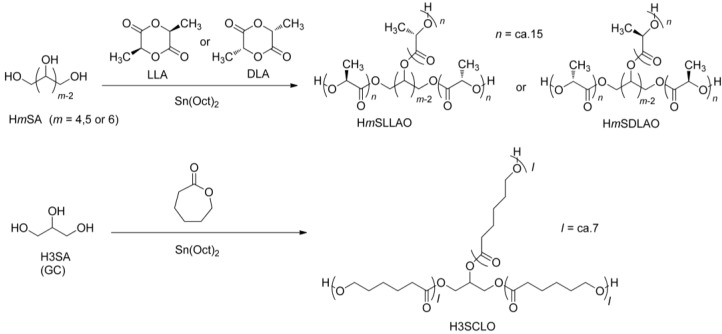
Synthesis of H*m*SLLAO, H*m*SDLAO and H3SCLO (*m* = 4, 5 or 6).

**Scheme 3:**
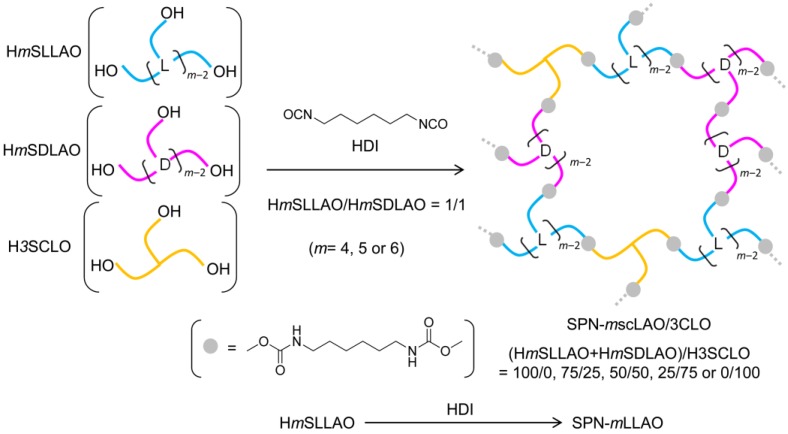
Synthesis of SPN-*m*scLAO/3CLO and SPN-*m*LLAO (*m* = 4, 5 or 6).

In this study, the crystallization behavior, thermal and mechanical properties of sugar-alcohol-based polymer networks (SPN-*m*scLAO/3CLO, *m* = 4, 5 or 6) prepared by the crosslinking reactions of ET-, XL- or SB-based *m*-armed star-shaped l-lactide and d-lactide oligomers (H*m*SLLAO and H*m*SDLAO), a GC-based 3-armed star-shaped ε-caprolactone oligomer (H3SCLO) and HDI at the weight ratios of H*m*SLLAO/H*m*SDLAO = 1/1 and (H*m*SLLAO + H*m*SDLAO)/H3CLO = 100/0, 75/25, 50/50, 25/75 or 0/100) were systematically investigated by comparing with those of the corresponding sugar-alcohol-based homochiral polymer network (SPN-*m*LLAO, *m* = 4, 5 or 6) prepared by the reaction of H*m*SLLAO and HDI (Scheme 2 and Scheme 3). Our attention is focused on the influence of the arm number on the crystallization behavior, thermal and mechanical properties.

## 2. Experimental Section 

### 2.1. Materials and Reagents

Glycerol (GC), *meso*-erythritol (ET), xylitol (XL), sorbitol (SB) and 1,2-dichloroethane (DCE) were purchased from Kanto Chemical Co., Inc. (Tokyo, Japan). Hexamethylene diisocyanate (HDI) and ε-caprolactone (CL) were purchased from Tokyo Chemical Industry Co., Ltd. (Tokyo, Japan). Chlorobenzene was purchased from Wako Pure Chemical Industries, Ltd. (Osaka, Japan). Tin (II) bis(2-ethylhexanoate) (Sn(Oct)_2_) was purchased from KISHIDA CHEMICAL Co., Ltd. (Osaka, Japan). l-Lactide (LLA, optical purity > 99%) and d-lactide (DLA, optical purity > 99%) were purchased from Musashino Chemical Laboratory, Ltd. (Tokyo, Japan). All the reagents were used without further purification.

### 2.2. Syntheses of HmSLLAO and HmSDLAO (m = 4, 5 or 6)

*m*-Armed star-shaped l-lactide and d-lactide oligomers (H*m*SLLAO and H*m*SDLAO, *m* = 4, 5 or 6) were synthesized modifying the synthetic method of H*m*SLLAO previously reported by Hao et al. [18] A typical synthetic procedure of H4SLLAO in this study is as follows: XL (0.859 g, 5.64 mmol), LLA (29.1 g, 202 mmol) and chlorobenzene (50 mL) were put into a nitrogen purged three-necked flask. The mixture was heated to 150 °C, and then Sn(Oct)_2_ (0.30 g, 1 wt % of the total weight of XL and LLA) was added into the flask. The resulting mixture was stirred at 150 °C under a nitrogen atmosphere for 24 h. The cooled reaction mixture was added to hexane (200 mL) with stirring, and then the supernatant was decanted off. This process was three times repeated and the separated precipitate was filtered, dried at 80 °C in a vacuum oven to give H4SLLAO as a white powder (yield: 29.8 g, 97%). The degree of polymerization (*n*) of lactate (i.e., a half of lactide) per arm measured by ^1^H-NMR method was 15.7. Similarly, H*m*SLLAOs and H*m*SDLAOs (*m* = 4, 5 or 6) other than H4SLLAO were synthesized by the ring-opening polymerizations of LLA and DLA with ET, XL or SB. The feed molar ratios, yields, *n* values, number- and weight average molecular weights (*M*_n_ and *M*_w_) measured by GPC method are summarized in Table 1. 

### 2.3. Synthesis of H3SCLO

3-Armed star-shaped ε-caprolactone oligomer (H3SCLO) was synthesized modifying the synthetic method of H3SCLO previously reported by Xie et al. [19] A typical synthetic procedure of H3SCLO in this study is as follows: GC (2.43 g, 26.4 mmol) and CL (57.6 g, 505 mmol) were put into a nitrogen purged three-necked flask. The mixture was heated to 150 °C, and then Sn(Oct)_2_ (0.060 g, 0.1 wt % of the total weight of GC and CL) was added into the flask. The resulting mixture was stirred at 150 °C under a nitrogen atmosphere for 24 h. The cooled reaction mixture was added to hexane (200 mL) with stirring, and then the supernatant was decanted off. This process was three times repeated and the separated precipitate was filtered, dried at 40 °C in a vacuum oven to give H3SCLO as a white powder (yield: 58.7 g, 98%). The synthetic data, degrees of polymerization (*n*) of caprolactone unit per arm, *M*_n_ and *M*_w_ are also summarized in Table 1.

### 2.4. Syntheses of SPN-mscLAO/3CLO and SPN-mLLAO (m = 4, 5 or 6)

Sugar-alcohol-based polymer networks (SPN-*m*scLAO/3CLO and SPN-*m*LLAO, *m* = 4, 5 or 6) were synthesized by crosslinking reactions of H*m*SLLAO/H*m*SDLAO/H3SCLO (the weight ratio of H*m*SLLAO/H*m*SDLAO = 1/1) and H*m*SLLAO with HDI, respectively. A typical synthetic procedure of SPN-5scLAO/3CLO 75/25 is as follows: A solution of H5SLLAO (1.71 g, 1.48 OH-mmol), H5SDLAO (1.71 g, 1.55 OH-mmol), H3SCLO (1.14 g, 1.32 OH-mmol) and HDI (0.40 g, 4.77 NCO-mmol) in DCE (40 mL) was poured into a petri dish (diameter: 97 mm) made of poly(tetrafluoroethylene). The molar ratio of OH/NCO in the mixture was fixed to 1/1.2. The mixture was dried at 60 °C for 24 h, and then 130 °C for 4 h in an electric oven. The obtained SPN-5scLAO/3CLO with the feed weight ratio of (H5SLLAO + H5SDLAO)/H3SCLO 75/25 (thickness: ca. 0.5 mm) was peeled off from the petri dish. SPN-*m*scLAO/3CLO (100/0, 75/25, 50/50, 25/75 and 0/100) films other than SPN-5scLAO/3CLO 75/25 were also prepared by a similar method. SPN-*m*scLAO/3CLO 100/0 and SPN-*m*scLAO/3CLO 0/100 were also abbreviated as SPN-*m*scLAO and SPN-3CLO, respectively. The reaction product (SPN-*m*LLAO, *m* = 4, 5 or 6) of H*m*SLLAO with HDI was also prepared by a similar method for comparison.

### 2.5. Characterization and Measurements 

Proton nuclear magnetic resonance (^1^H-NMR) spectra were recorded on a Bruker AV-400 (400 MHz) (Madison, WI, USA) or JEOL JNM-ECA500 (500 MHz) (Akishima, Tokyo, Japan) using CDCl_3_ and tetramethylsilane as a solvent and an internal standard, respectively. 

Gel permeation chromatography (GPC) was carried out at 40 °C on a Shimadzu LC9A GPC analysis apparatus equipped with two OHpak SB-806M HQ GPC columns (Showa Denko) and a refractive index (RI) detector. *N*,*N*-Dimethylformamide (DMF) was used as an eluent at a flow rate of 0.5 mL·min^−1^. Polystyrene standards with a narrow distribution of molecular weights (*M*_w_: 580~377,400) were used for molecular weight calibrations.

Fourier transform infrared (FT-IR) spectra were recorded at room temperature in the range from 4000 to 700 cm^−1^ on a Shimadzu (Kyoto, Japan) IRAffinity-1S by the attenuated total reflectance (ATR) method. The IR spectra were acquired using 50 scans at a resolution of 4 cm^−1^. 

Gel fraction was measured by the following procedure: A film (10 × 10 × 0.3–0.5 mm^3^) was dipped in chloroform at room temperature for 2 d, subsequently the film which was taken out was dried at 40 °C in a vacuum oven for 24 h. The gel fraction was calculated by the equation: Gel fraction (%) = 100 *w*_1_/*w*_0_; where *w*_0_ and *w*_1_ are the weights of original and dried films, respectively. 

X-ray diffraction (XRD) analysis of a film (10 × 10 × 0.3–0.5 mm^3^) on a glass cell was performed at ambient temperature on a Rigaku (Tokyo, Japan) RINT-2100 X-ray diffractometer at a scanning rate of 2.0°·min^−1^, using Cu Kα radiation (wavelength, λ = 0.154 nm) at 40 kV and 14 mA. All scans were in the range 5° ≤ 2*θ* ≤ 30° at a scanning rate of 1.0°·min^−1^ and a step size of 0.01°. 

Differential scanning calorimetry (DSC) measurements were performed on a Perkin-Elmer (Waltham, MA, USA) Diamond DSC in a nitrogen atmosphere. The as-prepared samples (5–8 mg) were heated from −100 °C to 200 °C at a heating rate of 20 °C·min^−1^, held at the temperature for 30 min to eliminate a thermal history of the sample, and then cooled to −100 °C at a cooling rate of 100 °C·min^−1^. After held at −100 °C for 3 min, the second heating scan was monitored at a heating rate of 20 °C·min^−1^. Glass transition temperature (*T*_g_), cold crystallization temperature (*T*_c,*x*_), enthalpy of cold crystallization (∆*H*_c,*x*_), melting temperature (*T*_m,*x*_) and enthalpy of melting (∆*H*_m,*x*_) for each component (*x* = LAO or CLO) was determined from the first and second heating curves. The crystallinities (*χ*_c,*x*_s) of homochiral (hc) or sc oligolactide crystallites and oligocaprolactone crystallites were calculated using the following equation:

$$\chi_{c,x}(\%)=\left(\frac{\Delta H_{m,x}}{w\Delta H_{m,x}^{0}}\right)\times 100$$

where *w* is the weight fraction of (H*m*SLLAO + H*m*SDLAO) and $\Delta H_{m,x}^{0}$ is enthalpy of 100% crystalline hc-PLA (93 J·g^−1^) or sc-PLA (142 J·g^−1^) in case of *x* = LAO [39], and *w* is the weight fraction of H3SCLO and $\Delta H_{m,x}^{0}$ is enthalpy of 100% crystalline PCL (139 J·g^−1^) in case of *x* = CLO [40]. 

Morphology of fractured surfaces of the conetworks was observed by field emission-scanning electron microscopy (FE-SEM), using a Hitachi S-4700 machine (Hitachi High-Technologies Corporation, Tokyo, Japan). All samples were fractured after immersion in liquid nitrogen for about 5 min. The fracture surfaces were sputter coated with gold to provide enhanced conductivity.

Polarized optical microscopy was performed on an Olympus BXP polarizing microscope equipped with a Japan High-tech hot-stage RH-350 and a Sony CCD-IRIS color video camera. After a sample was heated to 220 °C on the hot-stage and held at 220 °C for 30 min, it was cooled to a specified temperature (110–140 °C) at a cooling rate of 50 °C min^−1^ and the growing of spherulites was monitored over time at the temperature. 

Dynamic mechanical analysis (DMA) of the rectangular plates (40 × 8 × 0.3–0.5 mm^3^) was performed on a Rheolograph Solid instrument (Toyo Seiki Co., Ltd., Tokyo, Japan) under an atmosphere of air with a chuck distance of 20 mm, a frequency of 1 Hz and a heating rate of 2 °C min^−1^, based on ISO 6721-4:1994 (Plastics-Determination of dynamic mechanical properties, Part 4: Tensile vibration–Non-resonance method). 

The *x*% weight loss temperature (*T*_d*x*_, *x* = 5, 10 or 20) was measured on a Shimadzu TGA-50 thermogravimetric analyzer. A sample of about 5 mg was heated from room temperature to 500 °C at a heating rate of 20 °C·min^−1^ in a nitrogen purge stream at a flow rate of 50 mL·min^−1^. 

Tensile testing of rectangular specimens (length 45 mm, width 7 mm, thickness 0.3–0.5 mm) was performed at 25 °C using a Shimadzu Autograph AG-1 based on the standard method for testing the tensile properties of plastics (JIS K7161:1994 (ISO527-1:1993)). Span length and testing speed were 25 mm and 3 mm·min^−1^, respectively. Five specimens were tested for each set of samples, and the mean values and the standard deviation were calculated. 

## 3. Results and Discussion

### 3.1. Characterization of HmSLLAOs, HmSDLAOs and H3SCLO

**Figure 1:**
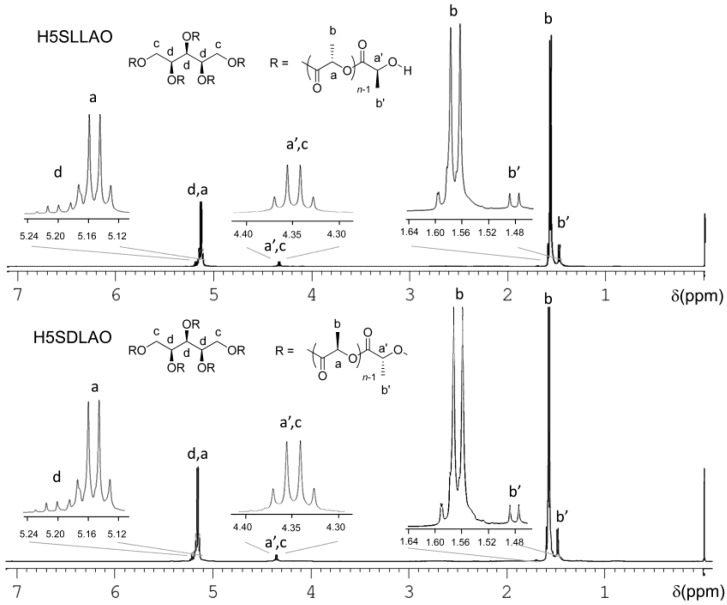
500 MHz ^1^H-NMR spectra of H5SLLAO and H5SDLAO in CDCl_3_.

H*m*SLLAO and H*m*SDLAO (*m* = 4, 5 or 6) were synthesized by the ring-opening polymerizations of LLA and DLA with ET, XL or SB, respectively (Scheme 1 and Scheme 2). The feed molar ratio of [LA]_0_/[OH]_0_ was optimized as the degree of polymerization per arm (*n*) by the ^1^H-NMR method becomes approximately 15, which is a minimum value to form sc-crystallites [36]. If the *n* value is much higher than 15, the subsequent crosslinking reaction with HDI becomes more difficult because of a lower hydroxy content. Figure 1 shows ^1^H-NMR spectra of H5SLLAO and H5SDLAO in CDCl_3_. Methine proton signals (H^a^ and H^a′^) of repeating and terminal lactate units of H5SLLAO were separately observed at δ 5.15 and 4.35 ppm, respectively. The two signals overlapped with the methine (H^d^) and methylene (H^c^) proton signals of the XL moiety, respectively. Additionally, methyl proton signals (H^b^ and H^b′^) of repeating and terminal lactate units of H5SLLAO were closely observed at δ 1.57 and 1.48 ppm, respectively. The *n* value of lactate unit (i.e., half of lactide) for H5SLLAO was calculated to be 15.7, based on the integral ratio of H^a,d^/H^a′,c^ = [5(*n* − 1)H + 3H]/(5H + 4H) = (5*n* − 2)/9. The *n* value measured by the ^1^H-NMR method for H5SLLAO was a little higher than the theoretical *n* value (14.3) calculated from the feed LLA/XL ratio, attributable to the removal of a slight amount of low-molecular-weight oligomers during the repeated decantation with hexane. As is shown in Figure 1, the ^1^H-NMR spectrum of H5SDLAO was similar to that of H5SLLAO. The observed *n* value of H5SDLAO was 14.9, which was also a little higher than the theoretical value (13.3). The molecular weights calculated from the observed *n* values (15.7 and 14.9) of H5SLLAO and H5SDLAO were 5809 and 5521, respectively, which were a little higher than their *M*_n_ values (3750 and 3240) by the GPC method (Table 1), reflecting that the molecular size of a star-shaped polymer is smaller than that of the corresponding linear polymer. The ^1^H-NMR spectra of H*m*SLLAOs and H*m*SDLAOs (*m* = 4 and 6) are shown in Figures S1–S4 (see Supplementary Materials). The observed *n* values of H*m*SLLAO and H*m*SDLAO (*m* = 4 or 6) were 15.4 and 14.8 (*m* = 4), or 14.6 and 16.6 (*m* = 6), respectively (Table 1). The molecular weights of H*m*SLLAO and H*m*SDLAO (*m* = 4 or 6) calculated from the *n* values were 4561 and 4388 (*m* = 4), or 6495 and 7360 (*m* = 6), respectively. These values were used for calculation of their feed amounts for the reaction with HDI.

**Figure 2:**
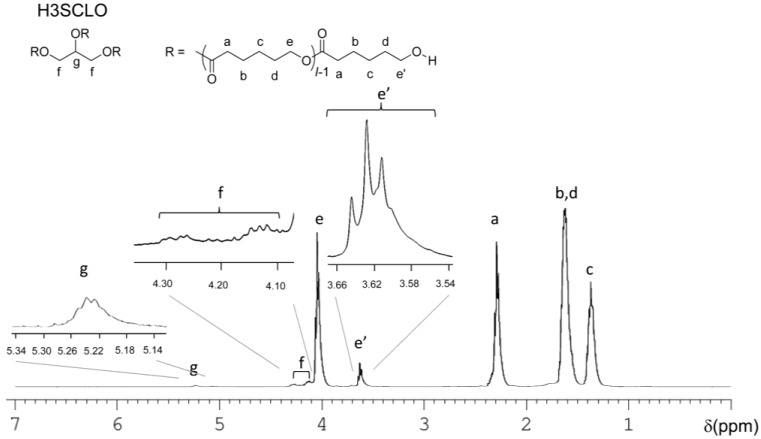
400 MHz ^1^H-NMR spectrum of H3SCLO in CDCl_3_.

H3SCLO was synthesized by the ring-opening polymerization of CL with GC at the [CL]_0_/[OH]_0_ ratio of 6.4. As the C-C and C-O bond number (6.4 × 7 + 1 = 45.8) per arm of H3SCLO is slightly higher than that (3 × 15 + 1 = 46) of H*m*SLLAO (or H*m*SDLAO), there is not much difference in arm length between H3SCLO and H*m*SLLAO (or H*m*SDLAO) under the assumption that both the oligomers are composed of extended chains. Figure 2 shows the ^1^H-NMR spectrum of H3SCLO in CDCl_3_. Oxygen-substituted methylene proton signals (H^e^ and H^e′^) of repeating and terminal caprolactone units of H3SCLO were separately observed at δ 4.05 and 3.62 ppm, respectively. The methylene (H^f^) proton signals of GC moiety were observed at 4.28 and 4.12 ppm, which partially overlapped with the H^e^ signal. The methine (H^g^) proton signal of GC moiety was observed at 5.24 ppm. The ^1^H-signals of other methylene protons (H^a^, H^b,d^ and H^c^) of caprolactone units were observed at 2.30, 1.63 and 1.39 ppm, respectively. The average *n* value of oligocaprolactone arms for H3SCLO was calculated to be 7.1, based on the integral ratio of H^e,f^/H^e′^ = [6(*n* − 1)H + 4H]/(6H) = (3*n* − 1)/3. The *n* value was a little higher than the theoretical *n* value (6.4), probably by the same reason for H5SLLAO. The molecular weight calculated from the observed *n* value of H3SCLO was 2523, which was also a little higher than the *M*_n_ (1560) by the GPC method (Table 1). The feed amount of H3SCLO for the reaction with HDI was calculation based on the molecular weight by the NMR method.

### 3.2. Characterization of SPN-mscLAO/3CLOs and SPN-mLLAOs

**Figure 3:**
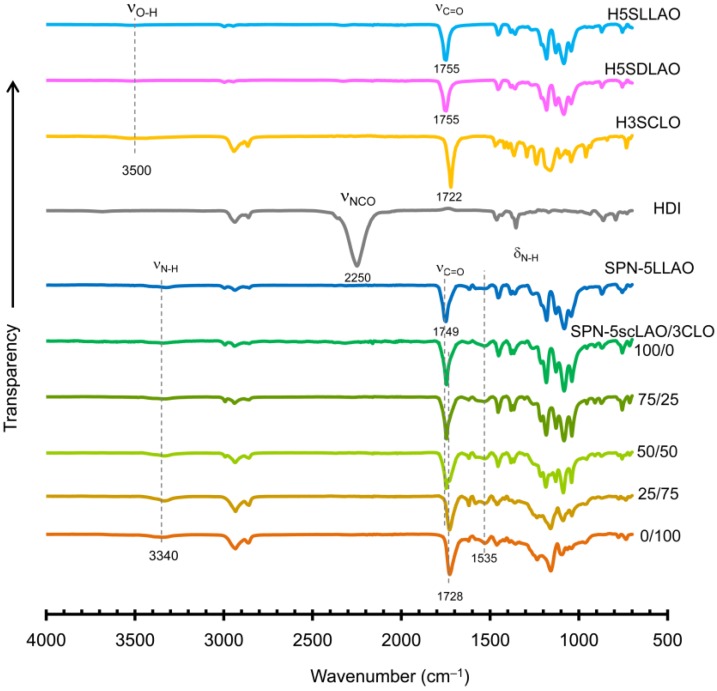
FT-IR spectra of H5SLLAO, H5SDLAO, H3SCLO, HDI, SPN-5LLAO and SPN-5scLAO/3CLOs (100/0, 75/25, 50/50, 25/75 and 0/100).

SPN-*m*scLAO/3CLO films were prepared by the crosslinking reaction of H*m*SLLAO, H*m*SDLAO, H3SCLO and HDI at the weight ratios of H*m*SLLAO/H*m*SDLAO = 1/1 and (H*m*SLLAO + H*m*SDLAO)/H3CLO = 100/0, 75/25, 50/50, 25/75 or 0/100) (Scheme 3). For comparison, SPN-*m*LLAO films were prepared by the crosslinking reaction of H*m*SLLAO and HDI. The feed OH/NCO molar ratio was fixed to 1/1.2. Figure 3 shows the FT-IR spectra of SPN-5scLAO/3CLOs and SPN-5LLAO compared with those of H5SLLAO, H5SDLAO, H3SCLO and HDI. H5SLLAO, H5SDLAO and H3SCLO displayed a weak absorption band of O-H stretching vibration (ν_O-H_) at around 3500 cm^−1^. HDI displayed a strong absorption band due to N=C=O stretching vibration (ν_NCO_) at 2250 cm^−1^. The corresponding bands were not observed for SPN-5LLAO and SPN-5scLAO/3CLOs, and new absorption bands of N-H stretching vibration (ν_N-H_) and N-H bending vibration (δ_N-H_) appeared at 3340 and 1535 cm^−1^, respectively. H5SLLAO and H5SDLAO showed an ester C=O stretching vibration (ν_C=O_) band at 1755 cm^−1^, which was in a little higher wavelength region than that of H3SCLO at 1722 cm^−1^. Similarly, the peak top wavelengths (1749 cm^−1^) of the ν_C=O_ bands of SPN-5LLAO and SPN-5scLAO/3CLO 100/0 (that is SPN-5scLAO) were higher than that (1728 cm^−1^) of SPN-5scLAO/3CLO 0/100 (that is SPN-3CLO). Therefore, the peak top wavelength of the ν_C=O_ band for SPN-5scLAO/3CLO (75/25–25/75) conetworks slightly increased with increasing 3CLO fraction. These ν_C=O_ bands of SPN-5LLAO and SPN-5scLAO/3CLO (100/0–0/100) are mainly ascribed to the ester carbonyl groups, and contained urethane ν_C=O_ bands as shoulder peaks at a lower wavelength region. Almost the similar trend was observed for the FT-IR spectra of SPN-*m*scLAO/3CLOs (*m* = 4 and 6) and SPN-*m*LLAOs (*m* = 4 and 6) (see Supplementary Materials, Figures S5 and S6). These results indicate that the urethanization reaction of hydroxy and isocyanate groups certainly proceeded for all the SPN-*m*scLAO/3CLOs and SPN-*m*LLAOs (*m* = 4, 5 and 6). The fact that gel fractions of SPN-*m*scLAO/3CLO and SPN-*m*LLAO films dipped in chloroform for 2 days were from 97 to 100 % also supported the formation of the network structure by the urethanization reaction. 

### 3.3. Stereocomplex Crystallization Behavior of SPN-mscLAO/3CLOs

**Figure 4:**
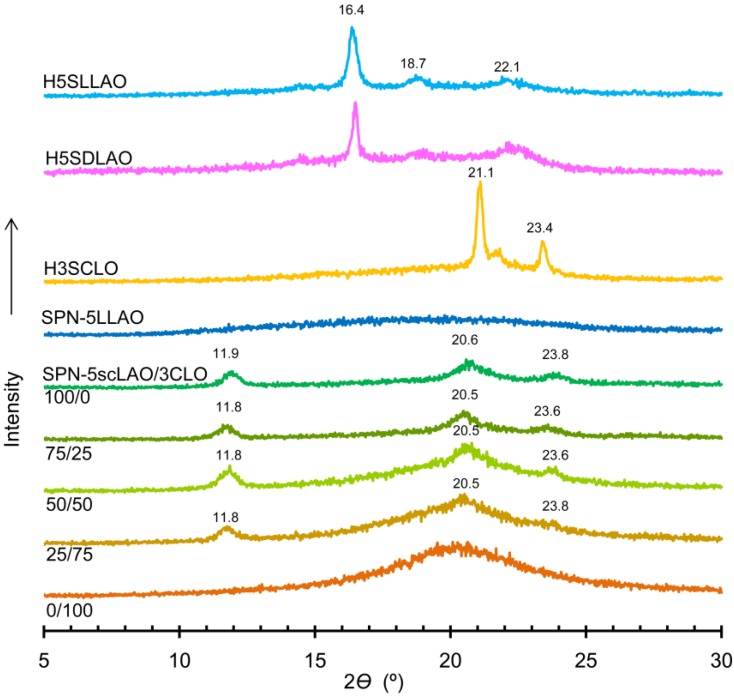
XRD patterns of H5SLLAO, H5SDLAO, H3SCLO, SPN-5LLAO and SPN-5scLAO/3CLOs (100/0, 75/25, 50/50, 25/75 and 0/100).

Figure 4 shows XRD profiles of H5SLLAO, H5SDLAO, H3SCLO, SPN-5LLAO and SPN-5scLAO/3CLOs. H5SLLAO and H5SDLAO exhibited diffraction peaks at 2*θ* values of 16.4, 18.7 and 22.1°characteristic of PLLA and PDLA crystallites, respectively [41]. H3SCLO displayed diffraction peaks at 2*θ* values at 21.3° and 23.6° characteristic of PCL crystallites [42]. SPN-5LLAO and SPN-5scLAO/3CLO 0/100 (that is SPN-3CLO) did not display such PLLA and PCL crystalline peaks, respectively, indicating that the homocrystallizations of the LLAO and CLO segments were prevented by the urethane crosslinkages. On the other hand, SPN-5scLAO/3CLOs exhibited diffraction peaks at 2*θ* values of 11.8–11.9°, 20.5–20.6° and 23.6–23.8°, characteristic of sc-LAO crystallites [39]. No diffraction peaks due to the PLLA and PDLA crystallites were observed in the XRD profiles of SPN-5scLAO/3CLOs, suggesting that sc crystallites were dominantly formed without any homo-crystallization. We could not determine whether the CLO segments slightly crystallized or not for SPN-5scLAO/3CLO conetworks, because the sc crystallization peaks at 20° and 24° were close to the CLO crystalline peaks at 21° and 23°. SPN*-m*LLAOs (*m* = 4 and 6) showed no crystalline peaks in a similar manner to SPN-5LLAO. Also, sc crystallites were dominantly formed for SPN*-m*scLAO/3CLOs 100/0, 75/25, 50/50 and 25/75 (*m* = 4 and 6) in a similar manner to SPN*-*5scLAO/3CLOs 100/0, 75/25, 50/50 and 25/75 (see Supplementary Materials, Figures S7 and S8). 

Table 2 summarizes the first and second heating DSC data for H3SCLO, SPN-3CLO, H*m*SLLAOs, H*m*SDLAOs, SPN-*m*LLAOs and SPN-*m*scLAOs. Also, Table 3 summarizes the first and second heating DSC data for SPN-*m*scLAO/CLOs. Additionally, the first and second heating DSC curves for H5SLLAO, H5SDLAO, H3SCLO, SPN-5LLAO and SPN-5scLAO/3CLOs (100/0, 75/25, 50/50, 25/75 and 0/100) are shown in Figure S9 (see Supplementary Materials). In the first heating curves at a rate of 20 °C·min^−1^, H5SLLAO and H5DLAO exhibited *T*_g_s at 41.1 and 49.2 °C, and *T*_m,LAO_s of homo-crystals at 139.0 and 136.0 °C, respectively. The crystallinities (χ_c,LAO_s) calculated from ∆*H*_m,LAO_s of H5SLLAO and H5DLAO were 23.4% and 28.3%, respectively. H3SCLO displayed a *T*_m,CLO_ at 55.9 °C, and showed no clear *T*_g_. Although we do not know its true reason, it is considered that the high crystallinity (χ_c,CLO_ = 43.6%) of H3SCLO makes the glass transition of amorphous segments less detectable. SPN-5LLAO displayed only a *T*_g_ at 58.2 °C, which was higher than those of H5SLLAO and H5SDLAO, attributable to the formation of urethane crosslinkages. The *T*_g_ (61.5 °C) of SPN-5scLAO was a little higher than that of SPN-5LLAO. Also, the *T*_m,LAO_ (187.5 °C) of SPN-5scLAO was much higher than that of H5SLLAO or H5SDLAO, indicating that sc-crystallites were dominantly formed for SPN-5scLAO. SPN-5scLAO/3CLO 75/25–25/75 conetworks had almost the same *T*_m,LAO_ as that of SPN-5scLAO. In the first heating DSC scan, the as-prepared SPN-3CLO displayed a *T*_c,CLO_ at −19.8 °C (∆*H*_c,CLO_ = −25.2 J·g^−1^) and a *T*_m,CLO_ at 29.3 °C (∆*H*_c,CLO_ = 27.0 J·g^−1^), suggesting that the original χ_c,CLO_ of SPN-3CLO is very low (1.4%) in agreement with the XRD result. In the second heating curves after cooled at a rate of 100 °C·min^−1^ from 200 °C, H5SLLAO and H5SDLAO displayed no *T*_m,LAO_s, suggesting that the homo-crystallization rate is very slow because the degree of polymerization is much lower than those of general PLLA and PDLA. On the other hand, SPN-5scLAO/3CLO 100/0–25/75 exhibited a *T*_c,LAO_ and *T*_m,LAO_, whose absolute ∆*H* values were almost comparable, indicating that sc-crystallites were regenerated during the second heating scan. However, the *T*_m,LAO_ and ∆*H*_m,LAO_ were slightly lower than those of the values obtained from the first heating scan. Although SPN-3CLO displayed a *T*_m,CLO_ at 33.0 °C, no cold crystallization peak was observed in the second heating curve, indicating that it crystallized during the cooling scan. This result suggests that the CLO segments have a higher crystallizability than the LAO segments. SPN-*m*scLAO/3CLOs100/0–25/75 (*m* = 4 and 6) also exhibited a similar sc-crystallization behavior to SPN-5scLAO/3CLOs 100/0–25/75. Concerning the influence of the arm number on the sc-crystallization behavior, it is interesting that the χ_c,LAO_s on the first and second heating DSC scans of SPN-4scLAO/3CLOs 100/0–50/50 were significantly lower than those of SPN-*m*scLAO/3CLOs 100/0–50/50 (*m* = 5 and 6), and that a higher order of the *T*_m,LAO_ on the first and second heating DSC scans for SPN-*m*scLAO/3CLOs100/0–25/75 was *m* = 5 > *m* = 6 > *m* = 4. Considering that the crystallization is disturbed by the crosslinking, the *T*_m,LAO_ should increase with decreasing *m* value. The fact that SPN-5scLAO/3CLOs exhibited higher *T*_m,LAO_s than SPN-4scLAO/3CLOs may be related to a higher conformational symmetry of XL than that of ET as is shown in Scheme 1. 

**Figure 5:**
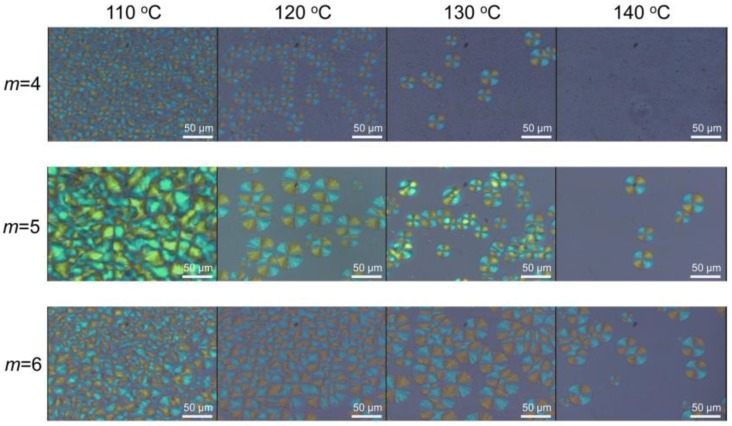
Polarized optical microscope images of the SPN-*m*scLAOs (*m* = 4, 5 and 6) held at a specified temperature for 10 min after melted at 220 °C.

**Figure 6:**
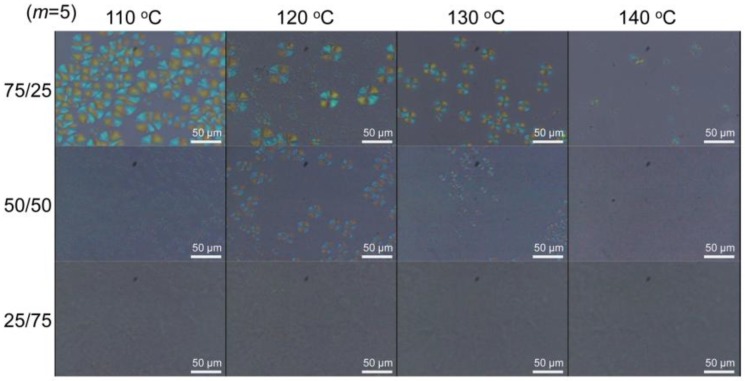
Polarized optical microscope images of the SPN-5scLAO/CLOs 75/25, 50/50 and 25/75 held at a specified temperature for 10 min after melted at 220 °C.

Figure 5 shows polarized optical microscope images of the SPN-*m*scLAOs (*m* = 4, 5 and 6) which were independently held at a specified temperature (110, 120, 130 or 140 °C) for 10 min after melted at 220 °C. For all of the SPN-*m*scLAOs, the crystallization temperature where the largest total volume of sc-LAO crystallites are formed was 110 °C, and the sc-LAO crystallization was suppressed with increasing temperature until 140 °C. When the crystallization temperatures are 110 and 120 °C, the larger order of the spherulite size was *m* = 5 > *m* = 6 > *m* = 4 in accordance with the higher order of *T*_m, LAO_ as above mentioned. When the crystallization temperatures are 130 and 140 °C, the larger order of the size and number of spherulites was *m* = 6 > *m* = 5 > *m* = 4. Figure 6 shows polarized optical microscope images of the SPN-5scLAO/3CLOs 75/25, 50/50 and 25/75 at the same condition as above. The size and number of spherulites decreased with increasing CLO fraction, indicating that the CLO segments disturbed the sc-crystallization. This result may be attributed to a possibility that the encounter between LLAO and DLAO segments may be made easier with decreasing CLO fraction. SPN-*m*scLAO/3CLOs 100/0–25/75 (*m* = 4 and 6) exhibited a similar trend to SPN-5scLAO/3CLOs 100/0–25/75 (see Supplementary Materials, Figures S10 and S11). 

### 3.4. Thermal and Mechanical Properties of SPN-mscLAO/3CLOs

**Figure 7:**
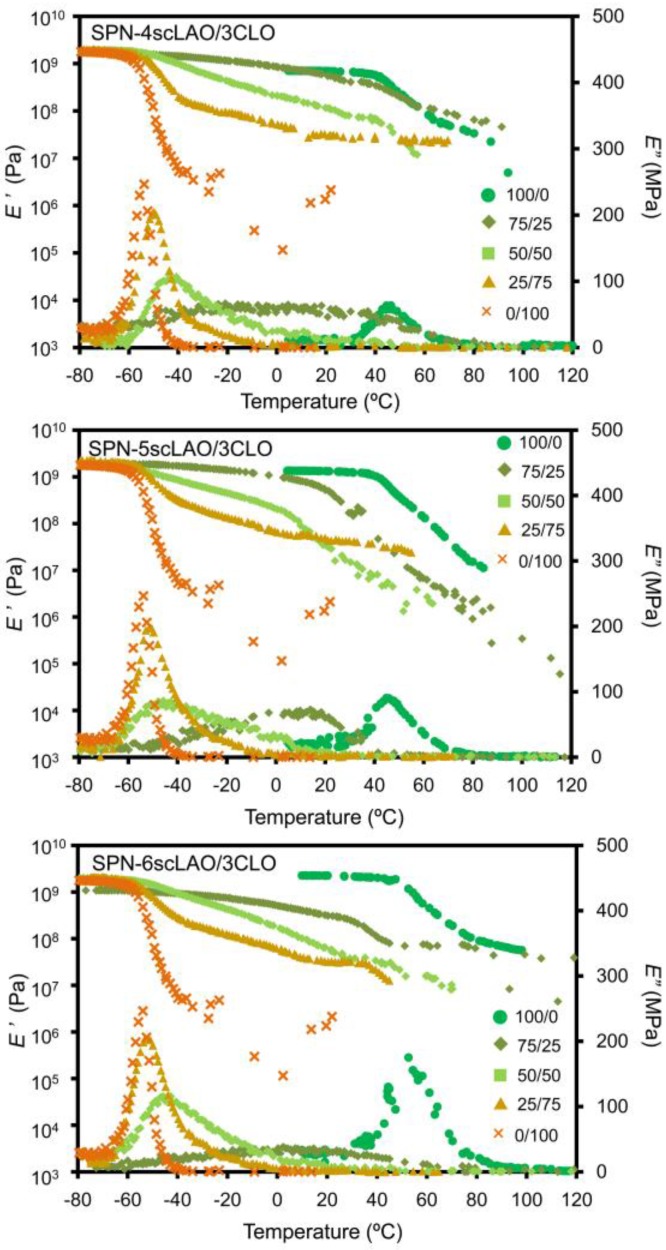
DMA curves of SPN-*m*scLAO/3CLOs 100/0, 75/25, 50/50, 25/75 and 0/100 (*m* = 4, 5 and 6).

Figure 7 shows DMA curves of SPN-*m*scLAO/3CLOs 100/0–0/100 (*m* = 4, 5 and 6). The loss modulus (*E*′′) peak temperatures related to the *T*_g_s for SPN-4scLAO, SPN-5scLAO and SPN-6scLAO were 44.5, 45.9 and 52.6 °C (Table 4), respectively, in agreement with the fact that their *T*_g_s obtained from the first heating DSC scans were 56.0, 61.5 and 65.4 °C (Table 2). This order accorded with the higher order of crosslinking density. The *E*′′ peak temperature of SPN-3CLO was −53.9 °C. All the conetworks (75/25–25/75) exhibited one *E*′′ peak temperature which decreased with increasing CLO fraction, reflecting that the LAO and CLO segments were compatibilized in agreement with the FE-SEM result (see Supplementary Materials, Figure S12). However, the difference of *E*′′ peak temperatures between the 25/75 and 0/100 samples was small, suggesting that the compatibility of the 25/75 samples is not good as compared with other samples. The storage modulus (*E*′) of SPN-4scLAO, SPN-5scLAO and SPN-6scLAO dropped at around 40, 41 and 47 °C due to a glass transition of the LAO-segment. The *E*′ of SPN-3CLO dropped at around −61 °C due to a glass transition of the CLO segment. Although the temperature where the *E*′ starts to drop for the conetworks decreased with increasing CLO fraction, the *E*′ reduction accompanied by the increasing temperature for SPN-4scLAO/3CLO 75/25 was gentle as compared with that for other 75/25 conetworks, reflecting that the *E*′′ peak was over a wide range of temperature. The broad *E*′′ peak of SPN-4scLAO/3CLO 75/25 may reflect an inhomogeneous distribution of the LAO and CLO segments. 

**Figure 8:**
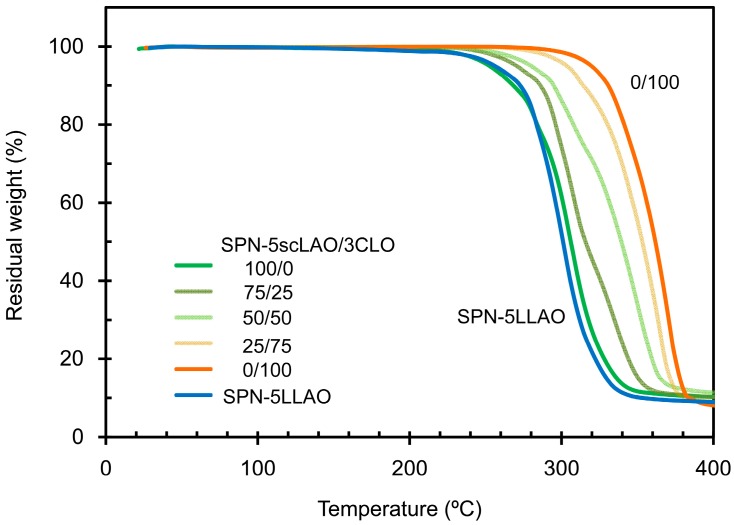
TGA curves of SPN-5scLAO/3CLOs (100/0, 75/25, 50/50, 25/75 and 0/100) and SPN-5LLAO.

Figure 8 shows TGA curves of SPN-5scLAO/3CLOs (100/0, 75/25, 50/50, 25/75 and 0/100) and SPN-5LLAO. As is obvious from the figure, the thermal degradation temperature of SPN-5scLAO/3CLO increased with increasing CLO fraction in agreement with the observation that *T*_d5_ (402 °C) of PCL is much higher than that (370 °C) of PLLA [43]. Also, there was no big difference between the TGA curves of SPN-5scLAO and SPN-5LLAO. The 5% weight loss temperatures (*T*_d*5*_s) and char yields at 400 °C for all the SPN-*m*scLAO/3CLOs and SPN-*m*LLAOs (*m* = 4, 5 and 6) are summarized in Table 4. Each of *T*_d5_s for all the SPN-*m*scLAO/3CLOs rose with increasing CLO fraction, and there was no big difference in *T*_d5_s and char yields among SPN-4scLAO/3CLO, SPN-5scLAO/3CLO and SPN-6scLAO/3CLO. SPN-*m*LLAOs exhibited slightly higher *T*_d5_s than SPN-*m*scLAOs (*m* = 4, 5 and 6). 

**Figure 9:**
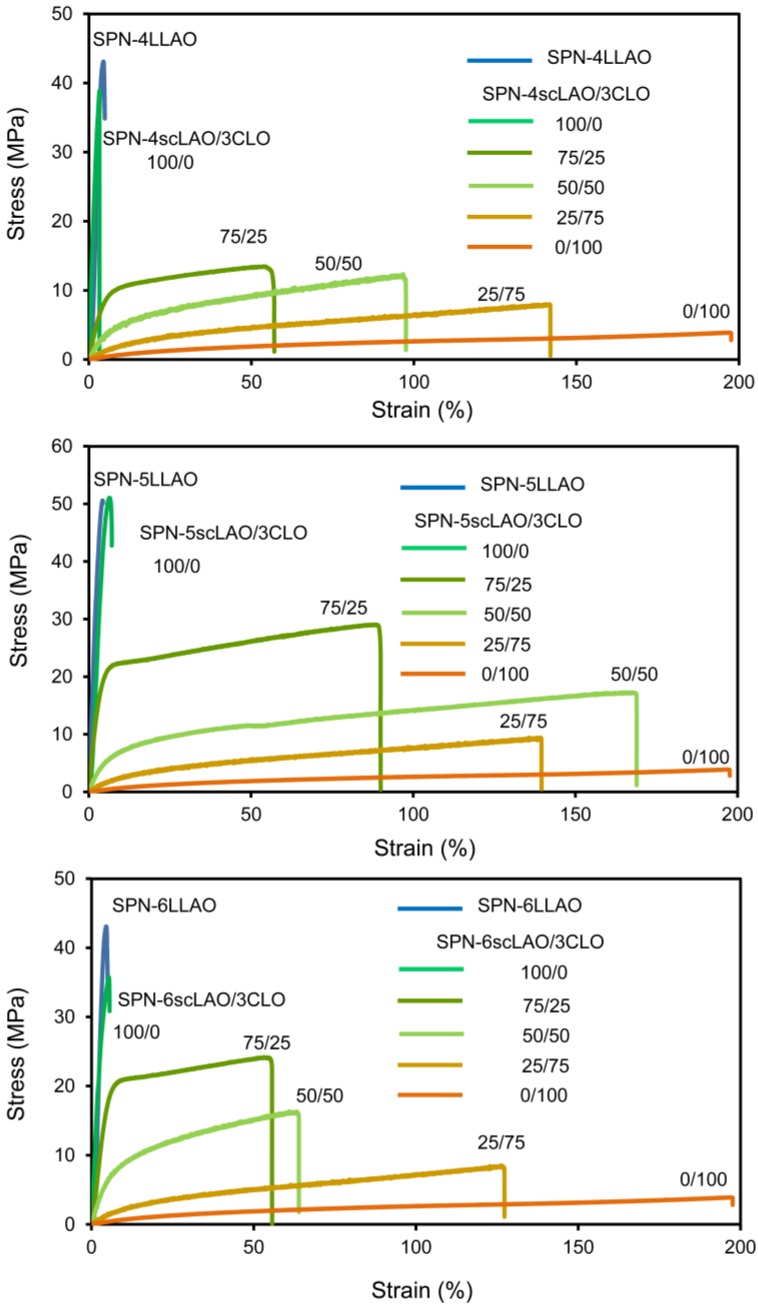
Stress-strain curves of SPN-*m*scLAO/3CLOs (100/0, 75/25, 50/50, 25/75 and 0/100) and SPN-*m*LLAOs (*m* = 4, 5 and 6).

Figure 9 shows typical stress-strain curves of SPN-*m*scLAO/3CLOs (100/0, 75/25, 50/50, 25/75, 0/100, *m* = 4, 5 and 6). There was little difference between the stress-strain curves of each SPN-*m*scLAO and SPN-*m*LLAO. Strain (elongation) at break and initial slope (tensile modulus) for each SPN-*m*scLAO were much lower and higher than those of SPN-3CLO, respectively, in agreement with the fact that LAO segment is much stiffer than CLO segment. Maximal stress and tensile modulus for the SPN-*m*scLAO/3CLOs (*m* = 4 and 6) decreased, and inversely elongation at break increased with increasing CLO fraction. For SPN-5scLAO/3CLO conetworks, the 50/50 sample exhibited a higher elongation at break than the 25/75 sample. As the toughness is defined as the energy needed to break a sample of unit area and unit length (J·m^−3^), it is given by the area under the stress-strain curve [37,38]. From the stress-strain curves shown in Figure 9, it is obvious that the toughnesses of SPN-5scLAO/3CLO 75/25 and 50/50 conetworks are much higher than those of other conetworks. 

**Figure 10:**
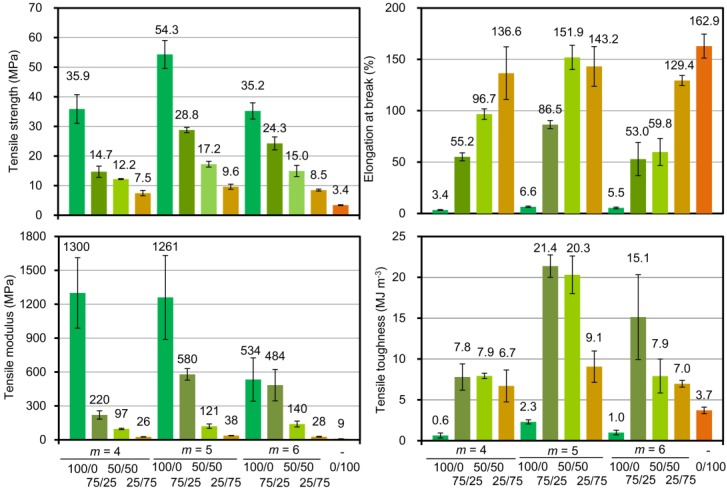
Tensile properties of SPN-*m*scLAO/3CLOs (100/0, 75/25, 50/50, 25/75 and 0/100, *m* = 4, 5 and 6).

Figure 10 shows tensile strength, modulus and elongation at break in addition to tensile toughness, which was calculated from the area of a stress-strain curve. When the SPN-*m*scLAO/3CLO 100/0–0/100 networks with the same arm number (*m*) were compared, the tensile strength and modulus decreased with increasing CLO fraction, and the elongation at break increased with increasing CLO fraction except that SPN-5scLAO/3CLO 50/50 displayed a little higher elongation at break than SPN-5scLAO/3CLO 25/75. Regarding the tensile toughness, the 75/25 or 50/50 conetwork exhibited the highest value. When the SPN-*m*scLAO/3CLO 100/0–25/75 networks with the same LAO fraction were compared, a higher order of tensile strength for SPN-*m*scLAO/3CLOs, *m* = 5 > *m* = 6 > *m* = 4. The networks with *m* = 5 displayed the highest elongation at break. As a result, the networks with *m* = 5 showed the highest tensile toughness. Among all the networks, SPN-5scLAO/3CLOs 75/25 and 50/50 exhibited the highest and second highest tensile toughnesses (21.4 and 20.3 MJ·m^−3^), respectively. We have already reported that the maximal tensile toughness and strength optimized for the conetworks (PEPN-4scLAO/4CLOs) prepared by the reactions of pentaerythritol-based 4-armed star-shaped LLA, DLA and CL with HDI were 25.8 MJ·m^−3^ and 18.5 MPa [44]. Although the tensile toughness of SPN-5scLAO/3CLO 75/25 was a little lower than that of PEPN-sc4LAO/4CLO 75/25, the tensile strength (28.8 MPa) of the former sample was much higher than that (18.5 MPa) of the latter sample.

## 4. Conclusions

As *m*-armed star-shaped biodegradable/biocompatible polyester networks possessing sugar-alcohol cores, SPN-*m*scLAO/3CLO and SPN-*m*LLAO (*m* = 4, 5 or 6) were synthesized, and the influence of arm number on the crystallization behavior, thermal and mechanical properties of were investigated. Stereocomplex (sc) crystallites are dominantly formed for SPN-*m*scLAO/3CLOs 100/0–25/75, whereas SPN-*m*LLAOs were amorphous. The higher order of melting temperature of sc-crystals for SPN-*m*scLAO/3CLOs 100/0–25/75 was *m* = 5 > *m* = 6 > *m* = 4. The sc-crystallinity of SPN-4scLAO/3CLOs 100/0–50/50 was significantly lower than those of SPN-*m*scLAO/3CLOs 100/0–50/50 (*m* = 5 and 6). The larger order of the sc-spherulite size at the crystallization temperature of 110 °C was *m* = 5 > *m* = 6 > *m* = 4 for SPN-*m*scLAO/3CLO 100/0. The size and number of sc-spherulites decreased with increasing crystallization temperature over the range of 110–140 °C and with increasing CLO fraction. Among all the networks, SPN-5scLAO/3CLOs 75/25 and 50/50 exhibited the highest and second highest tensile toughnesses (21.4 and 20.3 MJ·m^−3^), respectively. Especially, SPN-5scLAO/3CLO 75/25 is a promising polyester conetwork having a high *T*_m,LAO_, superior tensile strength and toughness usable for biomaterials.

## Figures and Tables

**Table 1:** The feed molar ratios, yields and *n* values of H*m*SLLAOs, H*m*SDLLAOs and H3SCLO.

Sample	Initiator	Feed molar ratio of LLA(DLA)/OH or CL/OH	Yield (%)	Theo. *n*	Obs. n *^1^	*M*_n_ *^2^	*M*_w_ *^2^
H3SCLO	GC	19.1	98	6.4	7.1	1560	2870
H4SLLAO	ET	28.0	94	14.0	15.4	2970	5100
H4SDLAO	ET	28.0	94	14.0	14.8	2900	4910
H5SLLAO	XL	35.8	99	14.3	15.7	3750	6430
H5SDLAO	XL	33.3	98	13.3	14.9	3240	6030
H6SLLAO	SB	36.3	97	12.1	14.6	4590	7210
H6SDLAO	SB	40.2	93	13.4	16.6	4720	7890

*^1^ The *n* values were measured by the ^1^H-NMR method; *^2^ The *M*_n_ and *M*_w_ values were measured by the GPC method.

**Table 2:** DSC data for H3SCLO, SPN-3CLO, H*m*SLLAOs, H*m*SDLAOs, SPN-*m*LLAOs and SPN-*m*scLAOs (*m* = 4, 5 and 6) *^1^.

Sample	First heating data				Second heating data			
	*T*_g_	*T*_m,*x*_ *^2^	∆*H*_m,*x*_ *^2^	χ_c,*x*_ *^2^	*T*_g_	*T*_m,x_*^2^	∆*H*_m,*x*_ *^2^	χ_c,*x*_ *^2^
	(°C)	(°C)	(J·g^−1^)	(%)	(°C)	(°C)	(J·g^−1^)	(%)
H3SCLO	-	55.9	60.6	43.6	-	38.4, 47.8	21.8, 41.1	45.3 *^3^
SPN-3CLO	−44.8	29.3	27.0	21.7	−43.8	33.0	32.8	26.3
H4SLLAO	36.6	132.4	16.8	18.1	36.1	-	0	0
H4SDLAO	41.2	132.1	17.3	18.6	46.7	-	0	0
H5SLLAO	41.1	139.0	23.4	25.2	50.7	-	0	0
H5SDLAO	49.2	136.0	28.3	30.4	38.0	-	0	0
H6SLLAO	-	135.4	29.9	32.1	34.5	-	0	0
H6SDLAO	-	130.3	23.2	25.0	31.4	-	0	0
SPN-4LLAO	62.3	-	0	0	26.4	-	0	0
SPN-5LLAO	58.2	-	0	0	28.9	-	0	0
SPN-6LLAO	67.0	-	0	0	31.4	-	0	0
SPN-4scLAO	56.0	175.0	19.3	14.8	43.9	-	0	0
SPN-5scLAO	61.5	187.5	31.0	23.7	35.8	173.0	29.4	22.5
SPN-6scLAO	65.4	187.0	31.5	24.3	31.4	170.9	30.6	23.6

*^1^ All the samples except for SPN-3CLO in the first heating scan, and SPN-5scLAO and SPN-6scLAO in the second heating scans did not exhibit cold crystallization peaks. The *T*_c,CLO_ and ∆*H*_c,CLO_ values for SPN-3CLO in the first heating scan were −19.8 °C and −25.2 J·g^−1^, respectively. The *T*_c,LAO_ and ∆*H*_c,LAO_ values for SPN-5scLAO in the second heating scan were 88.9 °C and −22.1 J·g^−1^, and those for SPN-6scLAO in the second heating scan were 88.8 °C and −27.1 J·g^−1^, respectively. *^2^ *T*_m,*x*_, ∆*H*_m,*x*_ and χ_c,*x*_ means DSC parameters for the component of *x* = CLO or LAO. *^3^ The total χ_c,CLO_ is divided into 15.7 and 29.6% for the two *T*_m,CLO_s at 38.4 and 47.8 ° C, respectively.

**Table 3:** DSC data for SPN-*m*scLAO/CLOs (*m* = 4, 5 and 6).

Sample		*T*_g_	*T*_m,CLO_	∆*H*_m,CLO_	χ_c,CLO_	*T*_c,LAO_	∆*H*_c,LAO_	*T*_m,LAO_	∆*H*_m,LAO_	χ_c,LAO_
		(°C)	(°C)	(J·g^−1^)	(%)	(°C)	(J·g^−1^)	(°C)	(J·g^−1^)	(%)
First heating data										
SPN-4scLAO/3CLO	75/25	-	-	-	0	-	0	177.7	19.6	20.2
	50/50	-	-	-	0	-	0	175.1	10.1	15.7
	25/75	-	-	-	0	-	0	176.1	6.23	19.6
SPN-5scLAO/3CLO	75/25	-	-	0	0	-	0	187.8	25.7	26.4
	50/50	-	-	0	0	-	0	187.8	15.8	24.5
	25/75	−41.2	17.3	5.4	5.7	-	0	187.8	5.64	17.6
SPN-6scLAO/3CLO	75/25	42.1	-	0	0	-	0	184.4	24.4	25.2
	50/50	−30.3	-	0	0	-	0	184.7	17.1	26.6
	25/75	−44.1	11.1	2.4	2.5	-	0	185.5	4.54	14.2
Second heating data										
SPN-4scLAO/3CLO	75/25	-	-	0	0	73.7	−16.1	168.4	11.5	11.8
	50/50	-	-	0	0	59.3	−8.18	170.1	11.6	18.1
	25/75	-	-	0	0	68.7	−2.78	173.4	2.43	7.6
SPN-5scLAO/3CLO	75/25	-	-	0	0	71.7	−18.4	173.4	21.4	22.0
	50/50	-	-	0	0	74.7	−8.00	181.8	10.8	16.8
	25/75	−39.0	32.0	5.5	5.8	92.9	−1.82	185.1	2.26	7.1
SPN-6scLAO/3CLO	75/25	27.7	-	0	0	66.9	−15.0	169.9	19.5	20.1
	50/50	−31.1	-	0	0	49.0	−3.88	171.6	3.1	4.9
	25/75	−40.9	28.6	6.7	7.1	82.8	−1.48	180.4	1.4	4.3

**Table 4:** *E*′′ peak temperatures of SPN-*m*scLAO/3CLOs (*m* = 4, 5 and 6) and TGA data for SPN-*m*LLAOs and SPN-*m*scLAO/3CLOs (*m* = 4, 5 and 6).

Sample		*E*′′ Peak temperature (°C)	*T*_d5_ (°C)	Char yield at 400 °C (%)
SPN-4LLAO		-	264	8.7
SPN-4scLAO/3CLO	100/0	44.5	255	4.5
	75/25	18.1	265	6.6
	50/50	−43.6	276	7.2
	25/75	−52.5	327	7.8
SPN-5LLAO		-	261	2.6
SPN-5scLAO/3CLO	100/0	45.9	254	10.2
	75/25	14.7	272	10.1
	50/50	−45.8	281	11.2
	25/75	−52.5	304	8.9
SPN-6LLAO		-	265	10.3
SPN-6scLAO/3CLO	100/0	52.6	254	9.2
	75/25	19.1	266	9.5
	50/50	−50.3	278	9.0
	25/75	−52.3	291	9.6
(SPN-3CLO)	0/100	−53.9	320	7.8

## Supplementary Materials

The following are available online at www.mdpi.com/2073-4360/9/11/582/s1, Figure S1: 500 MHz ^1^H-NMR spectrum of H4SLLAO in CDCl_3_, Figure S2: 500 MHz ^1^H-NMR spectrum of H4SDLAO in CDCl_3_, Figure S3: 400 MHz ^1^H-NMR spectrum of H6SLLAO in CDCl_3_, Figure S4: 400 MHz ^1^H-NMR spectrum of H6SDLAO in CDCl_3_,Figure S5: FT-IR spectra of H4SLLAO, H4SDLAO, H3SCLO, HDI, SPN-4LLAO and SPN-4scLAO/3CLOs 100/0, 75/25, 50/50, 25/75, 0/100, Figure S6: FT-IR spectra of H6SLLAO, H6SDLAO, H3SCLO, HDI, SPN-6LLAO and SPN-6scLAO/3CLOs 100/0, 75/25, 50/50, 25/75, 0/100, Figure S7, XRD patterns of H4SLLAO, H4SDLAO, H3SCLO, SPN-4LLAO and SPN-4scLAO/3CLOs 100/0, 75/25, 50/50, 25/75, 0/100, Figure S8: XRD patterns of H6SLLAO, H6SDLAO, H3SCLO, SPN-6LLAO and SPN-6cLAO/3CLOs 100/0, 75/25, 50/50, 25/75, 0/100, Figure S9: The first and second heating DSC curves for H5SLLAO, H5SDLAO, H3SCLO, SPN-5LLAO and SPN-5scLAO/3CLOs (100/0, 75/25, 50/50, 25/75 and 0/100), Figure S10: Polarized optical microscope images of the SPN-4scLAO/CLOs 100/0, 75/25, 50/50 and 25/75 held at a specified temperature for 10 min after melted at 220 °C, Figure S11: Polarized optical microscope images of the SPN-6scLAO/CLOs 100/0, 75/25, 50/50 and 25/75 held at a specified temperature for 10 min after melted at 220 °C, Figure S12: FE-SEM images of the fractured surfaces of SPN-*m*scLAO/3CLO 75/25–25/75 conetworks (*m* = 4, 5 and 6).
