# Supplementary material for: Stereocomplexation, Thermal and Mechanical Properties of Conetworks Composed of Star-Shaped l-Lactide, d-Lactide and ε-Caprolactone Oligomers Utilizing Sugar Alcohols as Core Molecules
Source: Polymers (Basel). 2017 Nov 6;9(11):582. doi: 10.3390/polym9110582 (PMC6418905; doi:10.3390/polym9110582)
Supplement: Supplementary file 1 [file polymers-09-00582-s001.pdf]

Stereocomplexation, thermal and mechanical properties of conetworks composed of star-shaped L-lactide, D-lactide and ε-caprolactone oligomers utilizing sugar alcohols as core molecules

Kaito Sugane, Hayato Takahashi, Toshiaki Shimasaki, Naozumi Teramoto and Mitsuhiro Shibata*

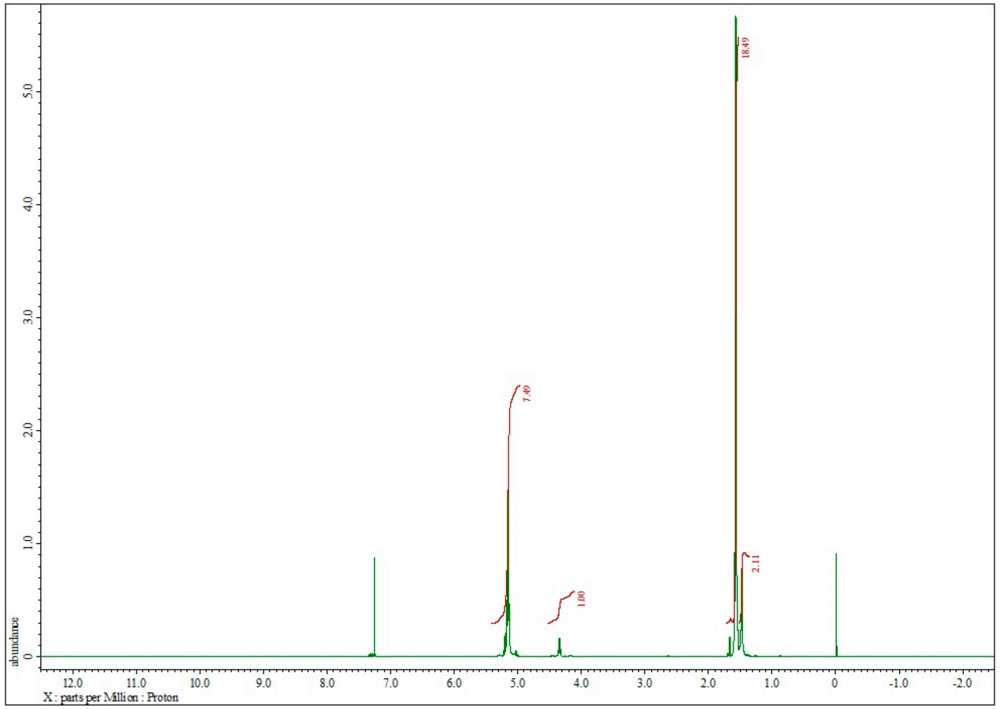

Figure S1. 500 MHz ^{1}H-NMR spectrum of H4SLLAO in $CDCl_3$.

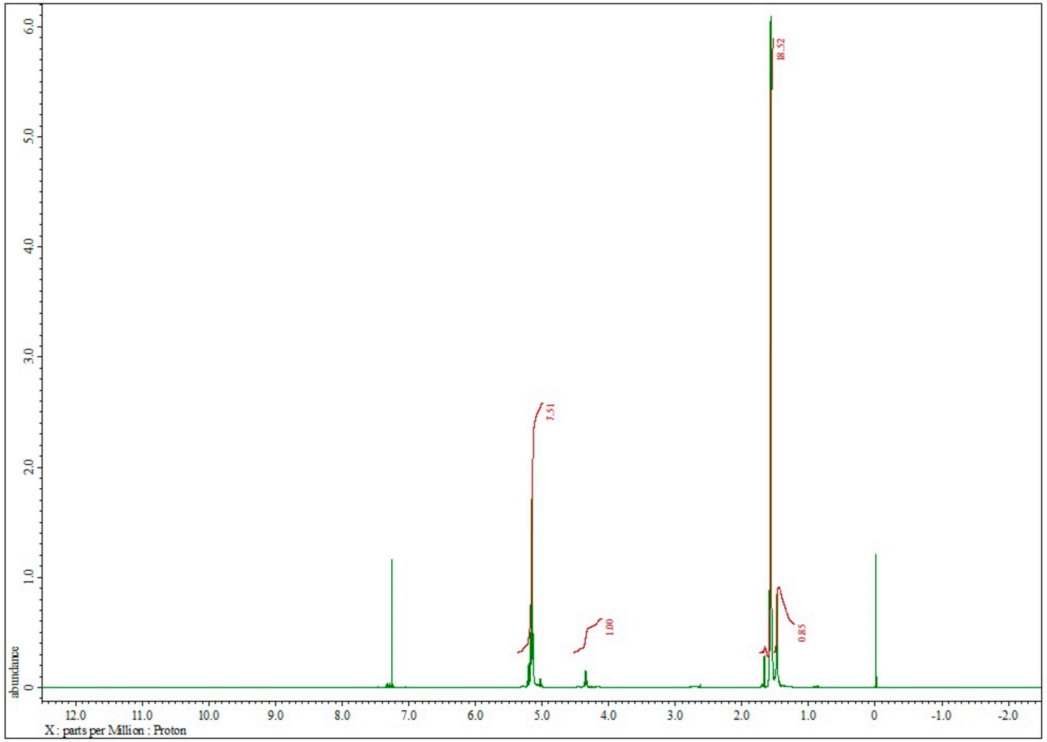

Figure S2. 500 MHz ¹H-NMR spectrum of H4SDLAO in $CDCl_3$.

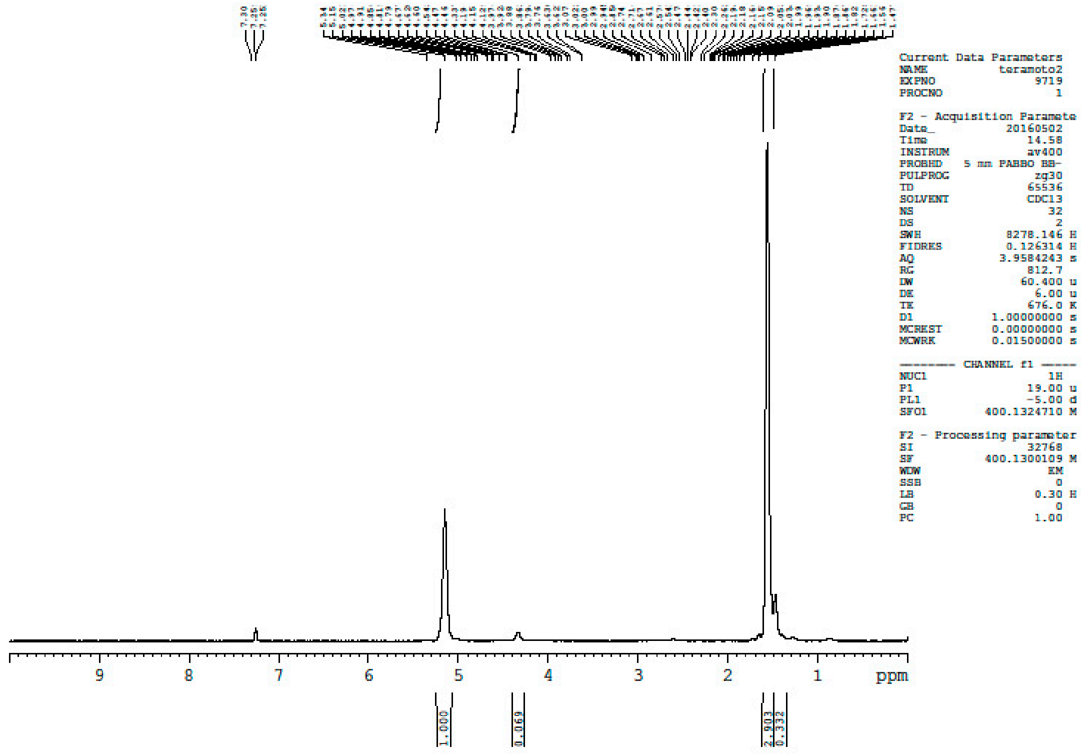

Figure S3. 400 MHz ^{1}H-NMR spectrum of H6SLLAO in $CDCl_3$.

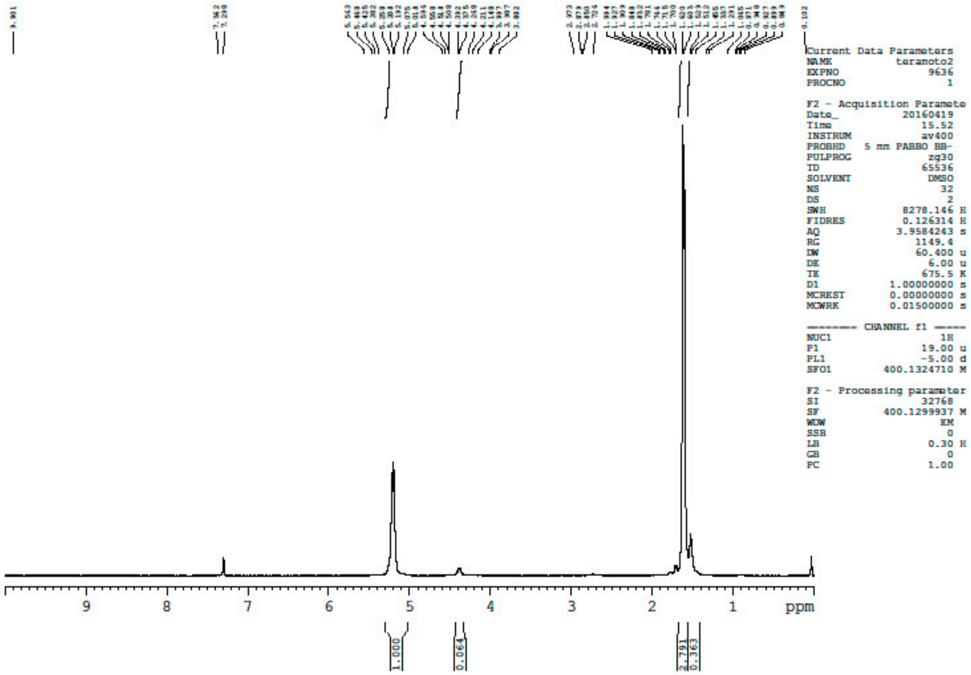

Figure S4. 400 MHz ¹H-NMR spectrum of H6SDLAO in $CDCl_3$.

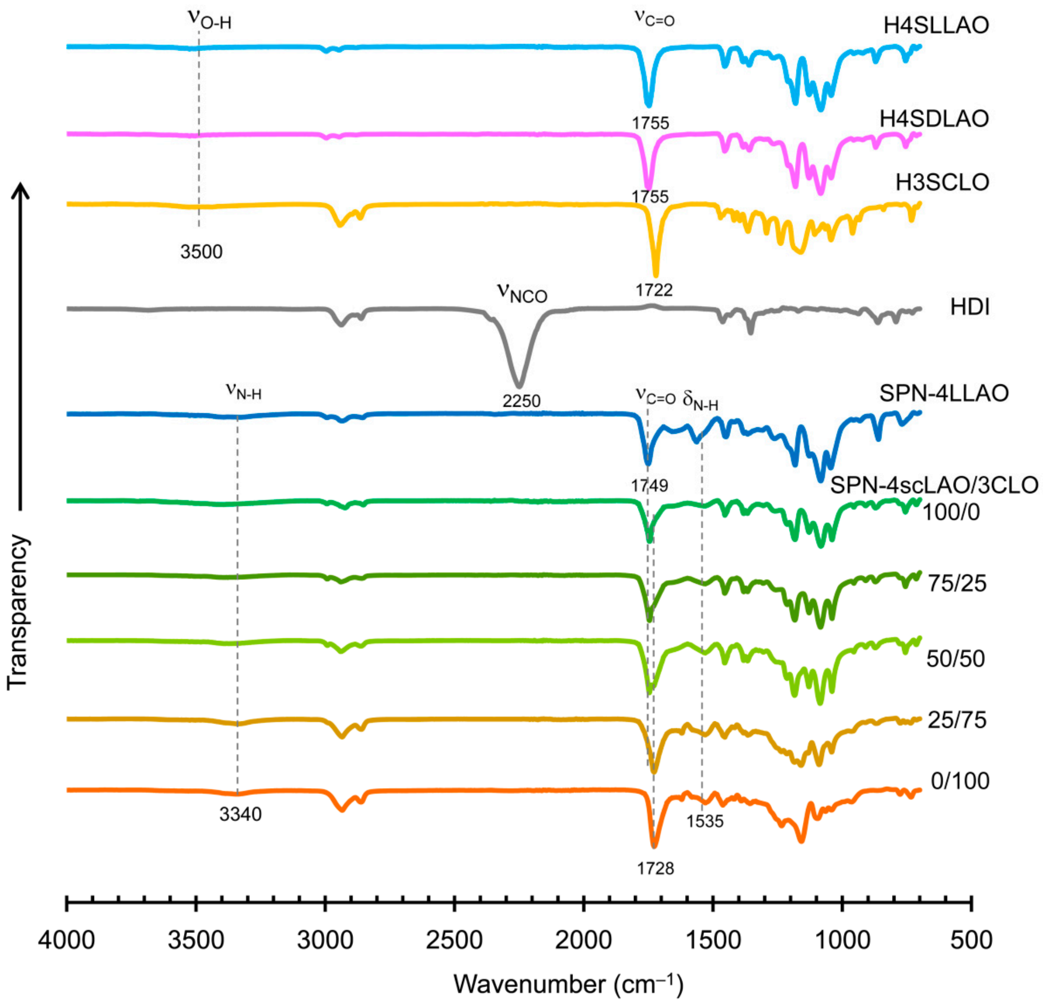

Figure S5. FT-IR spectra of H4SLLAO, H4SDLAO, H3SCLO, HDI, SPN-4LLAO and SPN-4scLAO/3CLOs (100/0, 75/25, 50/50, 25/75 and 0/100).

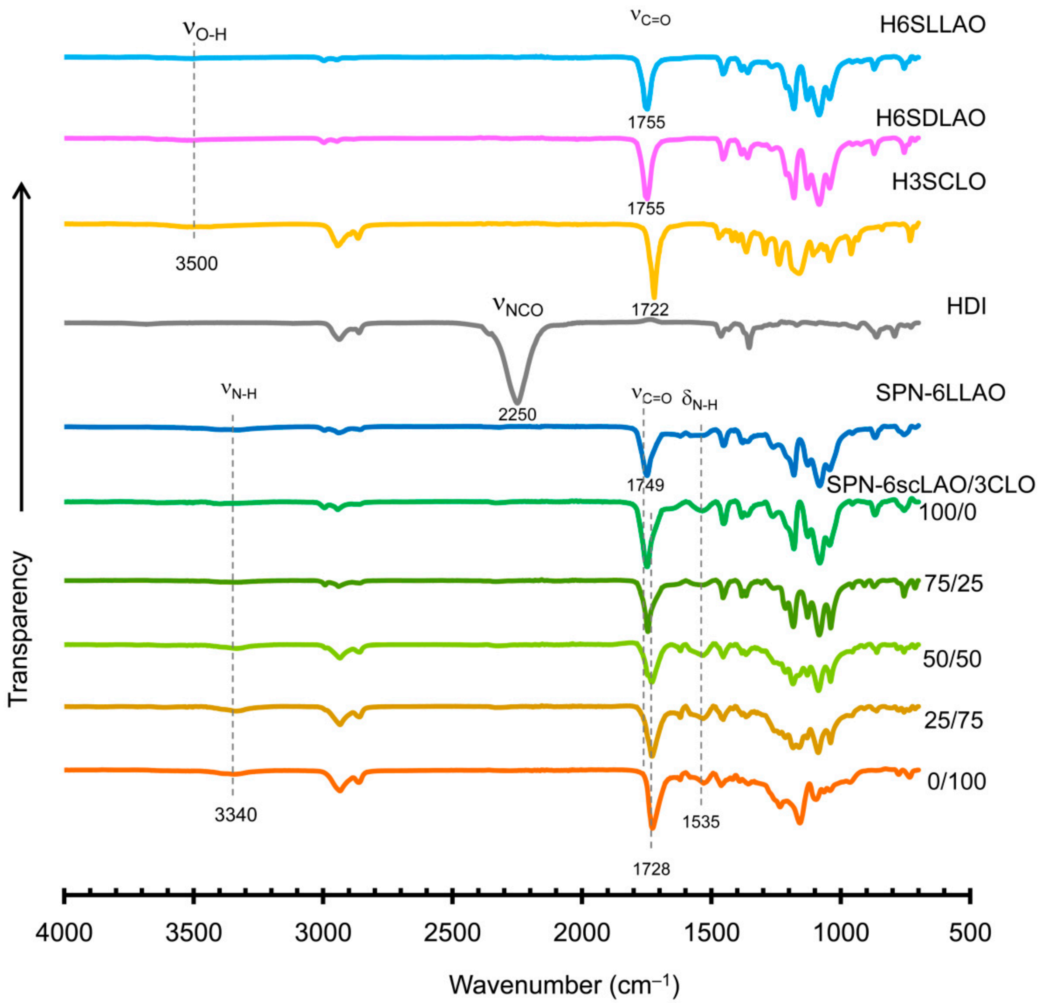

Figure S6. FT-IR spectra of H6SLLAO, H6SDLAO, H3SCLO, HDI, SPN-6LLAO and SPN-6scLAO/3CLOs (100/0, 75/25, 50/50, 25/75 and 0/100).

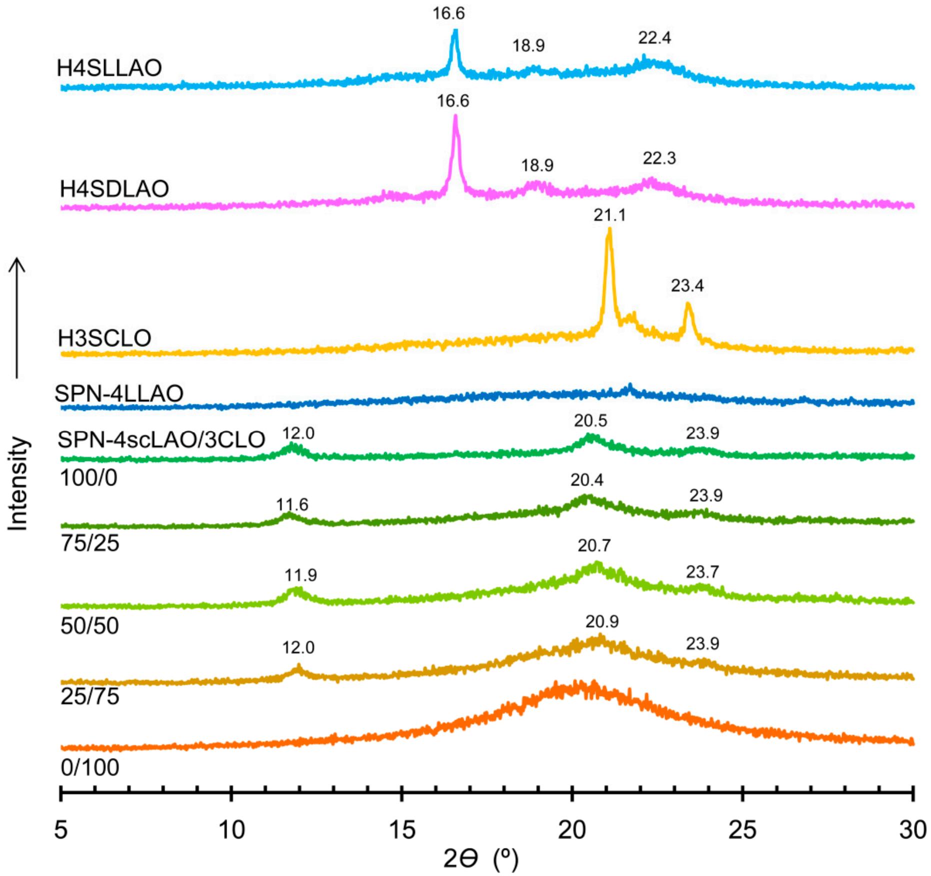

Figure S7. XRD patterns of H4SLLAO, H4SDLAO, H3SCLO, SPN-4LLAO and SPN-4scLAO/3CLOs (100/0, 75/25, 50/50, 25/75 and 0/100).

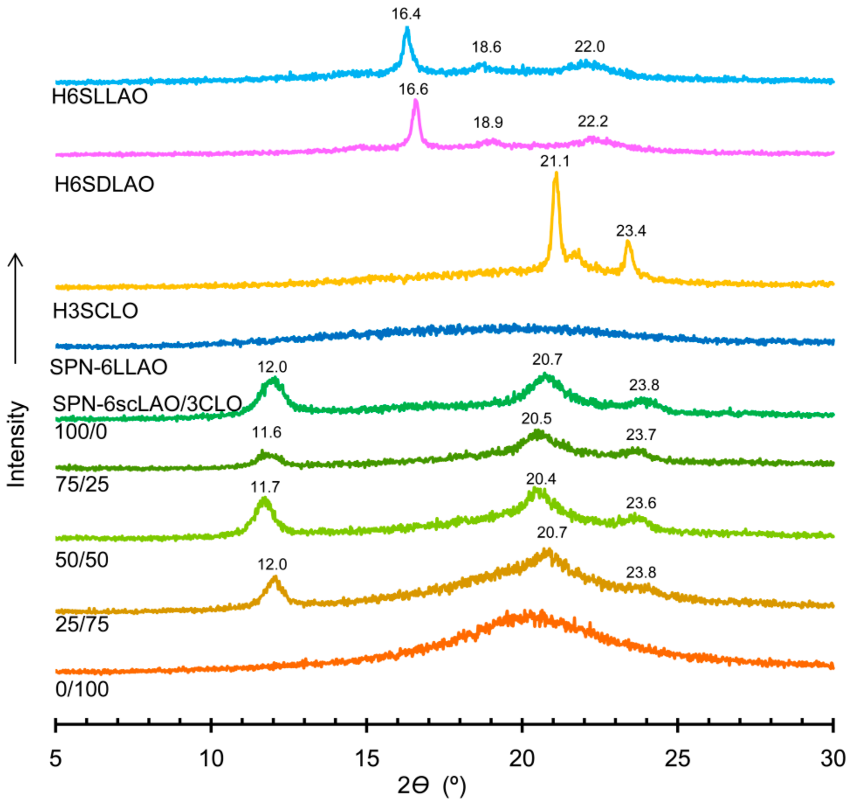

Figure S8. XRD patterns of H6SLLAO, H6SDLAO, H3SCLO, SPN-6LLAO and SPN-6scLAO/3CLOs (100/0, 75/25, 50/50, 25/75 and 0/100).

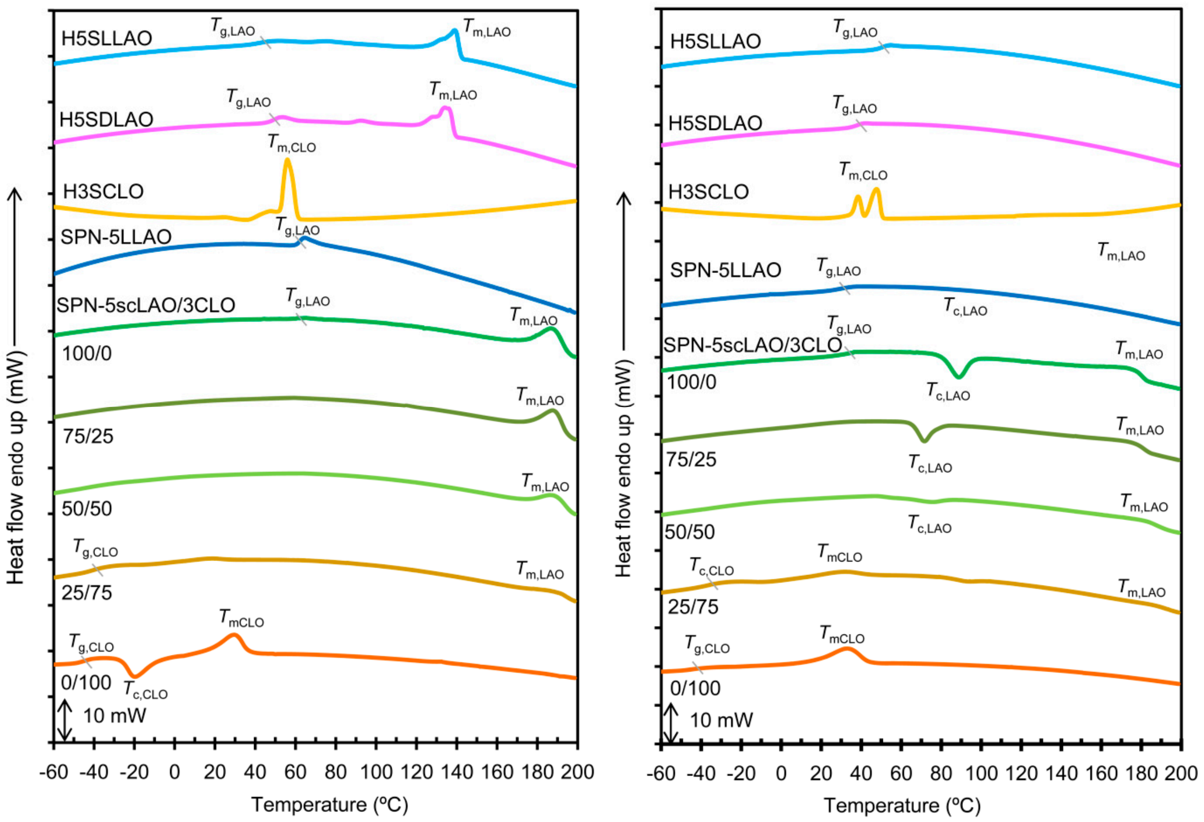

Figure S9. The first and second heating DSC curves for H5SLLAO, H5SDLAO, H3SCLO, SPN-5LLAO and SPN-5scLAO/3CLOs (100/0, 75/25, 50/50, 25/75 and 0/100).

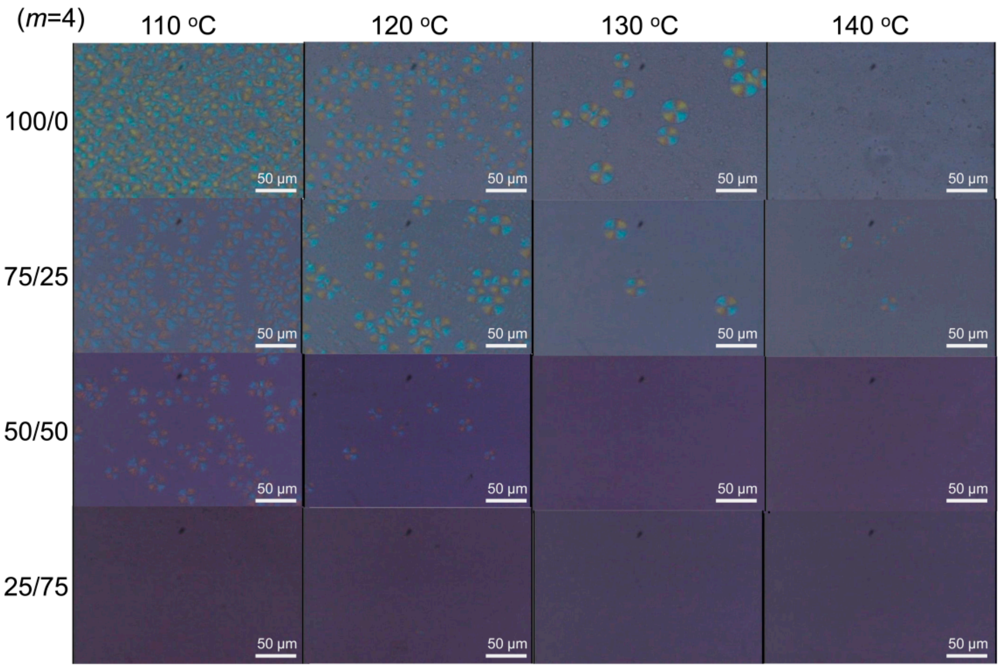

Figure S10. Polarized optical microscope images of the SPN-4scLAO/CLOs 100/0, 75/25, 50/50 and 25/75 held at a specified temperature for 10 min after melted at 220 °C.

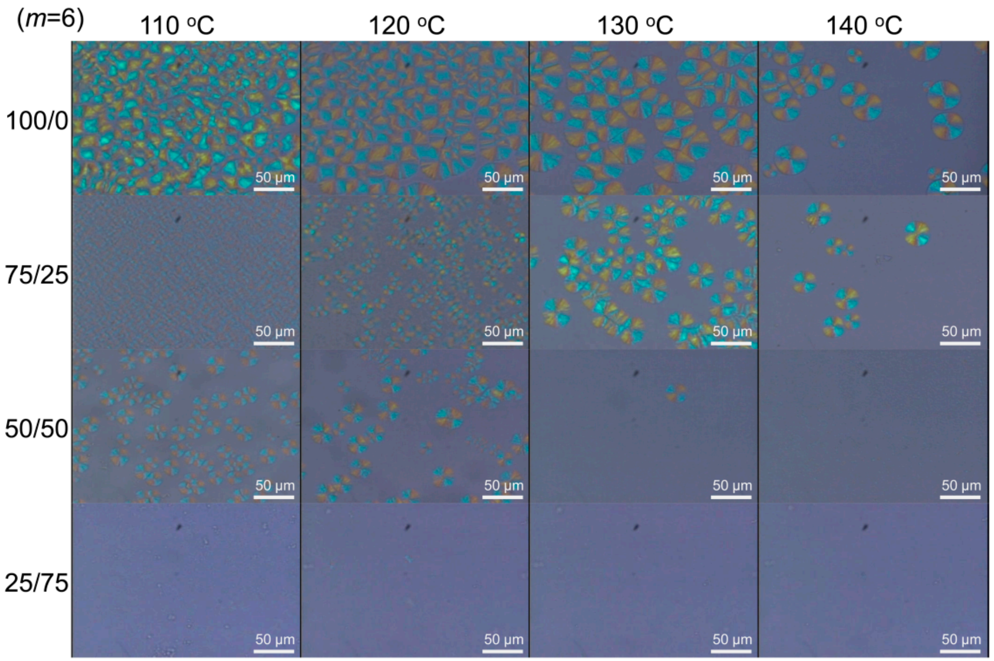

Figure S11. Polarized optical microscope images of the SPN-6scLAO/CLOs 100/0, 75/25, 50/50 and 25/75 held at a specified temperature for 10 min after melted at 220 °C.

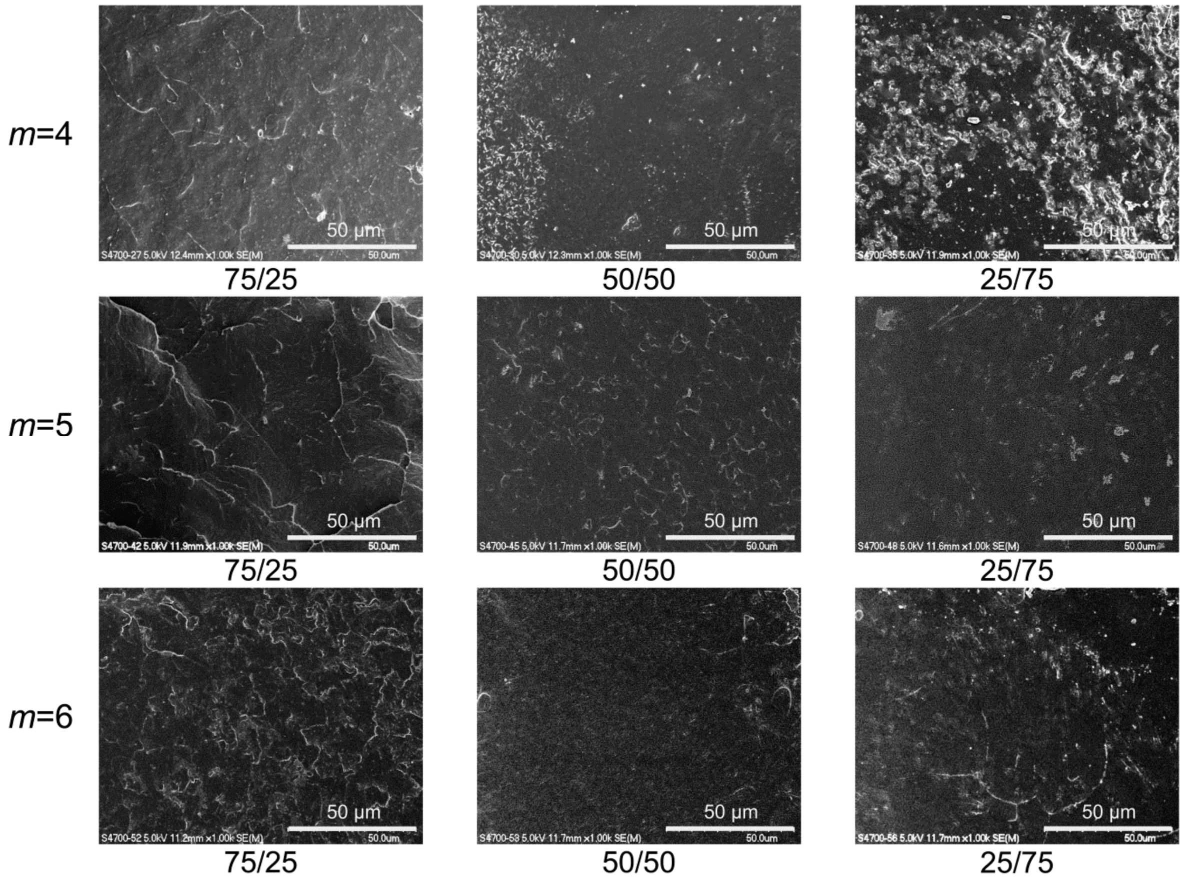

Figure S12. FE-SEM images of the fractured surfaces of SPN-*m*scLAO/3CLO 75/25-25/75 conetworks (*m* = 4, 5 and 6).
